# Genomic analysis of *Streptococcus pneumoniae* serogroup 20 isolates in Alberta, Canada from 1993–2019

**DOI:** 10.1099/mgen.0.001141

**Published:** 2023-11-28

**Authors:** Ashley N. Williams, Angela Ma, Matthew A. Croxen, Walter H. B. Demczuk, Irene Martin, Gregory J. Tyrrell

**Affiliations:** ^1^​ Department of Laboratory Medicine and Pathology, University of Alberta, 5-411 Edmonton Clinic Health Academy, Edmonton, Alberta, T6G 1C9 Canada; ^2^​ Alberta Precision Laboratory – Public Health Laboratory, 8440 112 Street NW, Edmonton, Alberta, T6G 2B7 Canada; ^3^​ Department of Pathology, University of Utah School of Medicine, 15 North Medical Drive East, Ste. #1100, Salt Lake City, UT, 84112 USA; ^4^​ Li Ka Shing Institute of Virology, University of Alberta, 6-010 Katz Centre for Health Research, 11315 - 87 Ave NW, Edmonton, Alberta, T6G 2E1 Canada; ^5^​ Women & Children’s Health Research Institute (WCHRI), University of Alberta, 5-083 Edmonton Clinic Health Academy (ECHA), 11405 87 Avenue NW Edmonton, AB, T6G 1C9 Canada; ^6^​ National Microbiology Laboratory, Public Health Agency of Canada, 1015 Arlington Street, Winnipeg, Manitoba, R3E 3R2 Canada

**Keywords:** Alberta, Global Pneumococcal Sequencing Project, invasive *Streptococcus pneumoniae*, sequencing Project, serotype 20B, ST235

## Abstract

In the province of Alberta, Canada, invasive disease caused by *Streptococcus pneumoniae* serogroup 20 (serotypes 20A/20B) has been increasing in incidence. Here, we characterize provincial invasive serogroup 20 isolates collected from 1993 to 2019 alongside invasive and non-invasive serogroup 20 isolates from the Global Pneumococcal Sequencing (GPS) Project collected from 1998 to 2015. Trends in clinical metadata and geographic location were evaluated, and serogroup 20 isolate genomes were subjected to molecular sequence typing, virulence and antimicrobial resistance factor mining, phylogenetic analysis and pangenome calculation. Two hundred and seventy-four serogroup 20 isolates from Alberta were sequenced, and analysed along with 95 GPS Project genomes. The majority of invasive Alberta serogroup 20 isolates were identified after 2007 in primarily middle-aged adults and typed predominantly as ST235, a sequence type that was rare among GPS Project isolates. Most Alberta isolates carried a full-length *whaF* capsular gene, suggestive of serotype 20B. All Alberta and GPS Project genomes carried molecular resistance determinants implicated in fluoroquinolone and macrolide resistance, with a few Alberta isolates exhibiting phenotypic resistance to azithromycin, clindamycin, erythromycin, tetracycline and trimethoprim-sulfamethoxazole, as well as non-susceptibility to tigecycline. All isolates carried multiple virulence factors including those involved in adherence, immune modulation and nutrient uptake, as well as exotoxins and exoenzymes. Phylogenetically, Alberta serogroup 20 isolates clustered with predominantly invasive GPS Project isolates from the USA, Israel, Brazil and Nepal. Overall, this study highlights the increasing incidence of invasive *S. pneumoniae* serogroup 20 disease in Alberta, Canada, and provides insights into the genetic and clinical characteristics of these isolates within a global context.

## Data Summary

The Alberta *S. pneumoniae* isolates included in this study (*n*=274) were uploaded to the NCBI SRA (https://www.ncbi.nlm.nih.gov/sra) and accession numbers are listed in Table S3, available in the online version of this article. *S. pneumoniae* genomes from the Global Pneumococcal Sequencing (GPS) project (*n*=95) were downloaded from the NCBI SRA on 28 October 2021, and accession numbers are listed in Table S3. The full-length *whaF* gene sequence was obtained from NCBI accession: CR931679.

**Impact Statement**

This study presents a comprehensive genomic characterization of invasive *S. pneumoniae* serogroup 20 identified in Alberta, Canada, which has been increasing in incidence since 2007. Included is a comparison of these isolates to invasive and non-invasive serogroup 20 isolates from the Global Pneumococcal Sequencing (GPS) Project, providing insights into the genetic diversity, virulence and antimicrobial resistance of serogroup 20 isolates from a global perspective. This study is aimed primarily at those engaged in infectious disease surveillance, epidemiology and genomics, and its findings may inform vaccine development and administration programmes. The study underscores the need for continuous monitoring and characterization of serogroup 20 isolates, given the increasing incidence and potential to cause severe disease.

## Introduction

*Streptococcus pneumoniae* is a Gram-positive bacterium that is a global healthcare concern. The natural reservoir of *S. pneumoniae* is the nasopharynx of asymptomatic carriers and is spread between individuals via respiratory droplets [1]. Pneumococci are a frequent cause of community-acquired pneumonia (CAP), as well as severe invasive pneumococcal disease (IPD). IPD manifests most often as bacteremia, meningitis or sepsis and is problematic primarily for children, the elderly, and those with comorbidities such as asthma, chronic lung/heart/kidney infections, alcohol abuse, cigarette smoking and diabetes [2–4]. Unfortunately, reduced susceptibility to antibiotics has emerged among clinical isolates, including to β-lactams, macrolides, lincosamides, tetracyclines, trimethoprim-sulfamethoxazole and fluoroquinolones, presenting treatment challenges [5].

*S. pneumoniae* isolates can be classified by their polysaccharide capsule of which there are currently over 100 recognized serotypes [6–8]. The invasiveness and type of disease caused by pneumococci varies between capsule types, with only a subset of serotypes having historically caused the bulk of IPD [9]. Among serotypes of clinical relevance is the relatively rare serotype 20, recently proposed to be renamed serogroup 20 [10, 11]. In comparison to more dominant serotypes, little is known about the epidemiological and clinical characteristics of serogroup 20; however, serogroup 20 isolates have been identified as both colonizers and agents of disease in children and adults, being associated with invasiveness and mortality [12–16]. In 2012, a renaming of serotype 20 to serogroup 20 was suggested following the discovery of two serotype 20 subgroups, which the authors designated as 20A and 20B [10, 11]. While indistinguishable by current serotyping antibodies used in diagnostics, serotypes 20A and 20B are structurally and genetically diverse, with truncation and loss of function of the capsular *whaF* gene being the hallmark genetic differentiator [10]. However, the discovering authors indicate that additional molecular work is required to confirm the connection between *whaF* truncation and the phenotypic differences between serotypes 20A and 20B [10].

In Canada, several vaccines are in use for the prevention of pneumococcal disease. Since 2000, the prevention of IPD in children in Canada and elsewhere using protein conjugate pneumococcal vaccines has been highly successful [17–24]. These protein conjugate vaccines include a limited set of prevalent serotypes and include a seven valent vaccine with serotypes 4, 6B, 9V, 14, 18C, 19F and 23F (PCV7), a 10 valent vaccine (PCV10; PCV7 serotypes plus 1, 5, 7F), and a 13 valent vaccine (PCV13; PCV10 serotypes plus 3, 6A and 19A) [25–27]. For the adult population, the principal pneumococcal vaccine that has been in widespread use is a 23 valent polysaccharide-based vaccine (PPV23; PCV13 serotypes plus 2, 9 N, 10A, 11A, 12F, 15B, 17F, 20, 22F and 33F) [28, 29]. Two new conjugate pneumococcal vaccines have recently been approved for adults in the USA and Canada. PCV15 includes the PCV13 serotypes plus serotypes 22F and 33F, and PCV20 includes PCV13 serotypes plus serotypes 22F, 33F, 8, 10A, 11A, 12F and 15B [30, 31]. The development of vaccines and the inclusion of additional serotypes continues to be key to the prevention of IPD. PPV23, despite covering more serotypes than conjugate vaccines, exhibits poor immunogenicity in infants under 2 years of age and lacks effectiveness in preventing community-acquired pneumonia in adults, as well as IPD in chronically ill patients and those over 75 [29, 32–34]. Other vaccines with broader serotype coverage are currently in development, including V116, a polyvalent pneumococcal conjugate vaccine that includes 3, 6A, 7F, 8, 9 N, 10A, 11A, 12F, 15A, 16F, 17F, 19A, 20, 22F, 23A, 23B, 24F, 31, 33F, 35B and 15B [35]. The serotypes in V116 include several that are not in currently licensed pneumococcal conjugate vaccines, but which contribute significantly to current adult pneumococcal disease burden, including serogroup 20.

Whole-genome sequencing (WGS) has become a valuable tool in the field of epidemiology, providing detailed genetic information about the organisms responsible for outbreaks [36]. By sequencing the entire genome of a pathogen, evolution and transmission patterns can be detected, as well as the carriage of virulence and antimicrobial resistance genes and the identification of sequence types (STs) of concern. The GPS Project (https://www.pneumogen.net/gps/index.html) is an example of a successful whole-genome surveillance effort [37]. The GPS Project, in collaboration with several contributors, has sequenced over 21 000 genomes of *Streptococcus pneumoniae* from various parts of the world (www.pneumogen.net/gps/gps-database-overview/; accessed 15Sept2023). The database contains metadata information for each isolate, which minimally includes date of collection and geography, but also allows for input of clinical data (e.g. gender, age, syndrome, source, HIV status, underlying conditions), serotype, sequence type and antimicrobial susceptibility testing results. Whole-genome analysis provides valuable information that can be used for tracking and preventing outbreaks, characterizing isolates of concern, and the development of therapies and vaccines.

Here, we report a cluster of IPD caused by serogroup 20 that began in 2007 and spiked in subsequent years in the province of Alberta, Canada. The objective of this work was to characterize 274 invasive serogroup 20 isolates in Alberta from 1993 to 2019 using genomics approaches and compare them to 95 invasive and non-invasive serogroup 20 isolates from the GPS Project from 1998 to 2015. Characterization of this minimally explored serogroup will foster a better understanding of the emerging invasive pathogen within Alberta, as well as provide a genomic overview of global serogroup 20.

## Methods

### Isolate and clinical data collection

Cases of IPD were defined as per the national case definition and are notifiable to Public Health in Alberta (population 4.36 million in 2019) [38]. Pneumococcal isolates from sterile sites are submitted to the Provincial Public Health Laboratory (PPHL) located in Edmonton, Alberta for serotyping and antimicrobial resistance profiling. Only one isolate was counted per case within a 30 day period unless the second isolate was a different serotype. Serogroup 20 isolates were not included in our analysis if there were duplicates collected from the same patient in the same year, the isolate did not grow for sequencing, the isolate had poor coverage as determined by Gubbins (see below), or the isolate had poor sequencing/assembly quality as determined by Quast (see below). Maps were obtained from https://open.canada.ca, which are licensed under the Open Government License – Canada and generated with Tableau Desktop v2021.2 and Inkscape v0.92.3.

### Laboratory identification, serotyping and antimicrobial susceptibility

*S. pneumoniae* isolates received at the PPHL were confirmed as *S. pneumoniae* based on characteristic morphology and optochin susceptibility [39]. All pneumococcal isolates that exhibited a positive Quellung reaction using commercial type-specific antisera obtained from Statens Serum Institut, Copenhagen, Denmark were assigned a serotype designation [40]. Antibiotic susceptibility was determined using the reference broth micro-dilution and disc diffusion methods as described by Clinical and Laboratory Standards Institute (CLSI) [41]. The following antimicrobial agents were assayed: amoxicillin, azithromycin, cefepime, cefotaxime, ceftriaxone, cefuroxime, chloramphenicol, clindamycin, ertapenem, erythromycin, induced clindamycin resistance, levofloxacin, linezolid, meropenem, moxifloxacin, penicillin, tetracycline, tigecycline, trimethoprim/sulfamethoxazole and vancomycin. All antibiotic powders were purchased from Sigma-Aldrich Canada (Oakville, Ontario). Interpretation of the MIC or disc diffusion (DD) tests were based on CLSI Performance Standards M100-Ed32 [42] and FDA breakpoints for tigecycline [43].

### Whole-genome sequencing and assembly

For samples collected in 2013 or earlier, DNA was extracted from cultures following standard protocol with Epicentre Masterpure Complete DNA and RNA Extraction Kit (Mandel Scientific, Guelph, ON). For samples collected after 2013, genomic DNA was extracted with a modified protocol for the MagaZorb DNA Mini-Prep Kit (Promega). Briefly, colonies were incubated overnight at 35 °C in Bacto Todd-Hewitt Broth (500 µl; BD Biosciences). Cultures were centrifuged at 6010 RCF for 2 min, supernatant removed, and cells resuspended in 12 mM Tris (500 µl). Centrifugation was repeated and cells resuspended in mutanolysin/hyaluronidase lysis solution [62 µl; 10 µl 3000 U ml^−1^ mutanolysin (Sigma), 2 µl 30 mg ml^−1^ hyaluronidase (Sigma) and 50 µl 10 mM Tris]. Lysozyme (15 µl, 100 mg ml^−1^; Sigma) was added and incubated for 1 h at 37 °C with shaking at 700 r.p.m. (Eppendorf ThermoMixer F1.5). Proteinase K solution (20 µl) and RNase A (20 µl, 20 mg ml^−1^; Qiagen or Invitrogen) were added and the tubes were incubated at room temperature for 5 min. ATL lysis buffer (200 µl) was added, and tubes incubated for 2 h at 56 °C with shaking at 900 r.p.m. (Eppendorf ThermoMixer F1.5). Extracts were centrifuged at 9391 RCF for 2 min, and wash, binding and elution steps were completed with the KingFisher ml Purification System (Thermo Scientific) with Qiagen Buffer EB. Genomes were sequenced either by NextSeq 500/550 or Illumina MiSeq with paired end reads (Illumina, San Diego, CA). The minimum and maximum genome coverages ranged from 77.6 and 99.7 % to the reference genome SRR3486078 (Alberta serogroup 20 ST235 isolate). Sequence reads from the Global Pneumococcal Sequencing Project for 95 serogroup 20 isolates were downloaded from the NCBI Sequence Read Archive (SRA) using the SRA Toolkit v 2.11.2 (https://github.com/ncbi/sra-tools). All genomes were assembled with Shovill v1.1.0 (https://github.com/tseemann/shovill) with ‘assembler’ set to ‘spades’, ‘gsize’ set to ‘2.2M’, and the ‘trim’ switch on. Annotations were performed with Bakta v1.2.2 (Bakta database v3.0) with ‘genus’ set to ‘Streptococcus’ and ‘gram’ to ‘+’ [44]. The quality of the assemblies was assessed with Quast 5.0.2 using BUSCO with switch ‘conserved-genes-finding’ on (Table S1) [45, 46].

### Bioinformatic analysis

Genomes were serotyped *in silico* using PneumoCaT v1.2.1 with default parameters and the top match for each genome was used even if it did not meet the default cut off values [47]. Subtyping of serogroup 20 genomes as 20A or 20B was determined by standalone blastn 2.12.0+ of the *whaF* gene that is part of the serogroup 20 capsular polysaccharide (CPS) cluster (NCBI accession: CR931679), where a full-length *whaF* suggests serotype 20B [10, 48]. WGS-based sequence typing was performed on assembled genomes with mlst v2.19 with default parameters and PubMLST [49] (https://github.com/tseemann/mlst). Global Pneumococcal Sequencing Clusters (GPSCs) were assigned using the ST Lookup Table found at https://www.pneumogen.net/gps/GPSC-ST.html. Antibiotic resistance genes were identified with the Resistance Gene Identifier (RGI) v5.2.0 with switches ‘include_loose’, ‘clean’ and ‘low_quality’ included, and ‘data’ set to ‘wgs’, which uses the Comprehensive Antibiotic Resistance Database (CARD) v3.1.4 [50]. Virulence factors were identified with Abricate v1.0.1 (https://github.com/tseemann/abricate) with the core Virulence Factor Database (VFDB) (DB version: 30 December 2021) [51]. The pangenome for Bakta annotated genomes was calculated using Anvi’o v7.1 with the workflow for microbial pangenomics, which implements the minbit parameter, DIAMOND, and MCL, and the results were visualized with ‘anvi-display-pan’ [52–56]. To use our Bakta annotated genomes .gbff files were initially processed with ‘anvi-script-process-genbank’ and these external files were used in the rest of the workflow, and ‘gene-caller’ ‘NCBI_PGAP’ was used during the genomes storage database creation step. Average nucleotide identity (ANI) was calculated with Anvi’o using FastANI with default parameters [57].

Phylogenetic relationships were assessed using core SNPs. Core SNPs were extracted from sequence reads and aligned with Snippy v4.6.0 using the assembled genome of SRR3486078 as a reference (https://github.com/tseemann/snippy) (Table S2). The core SNP alignment was cleaned to replace non-nucleotide characters with ‘N’ using ‘snippy-clean_full_aln’. Regions of recombination within the alignment were determined with Gubbins v3.1.4 using marginal ancestral reconstruction with IQ-TREE v2.0.3 and switches ‘tree-builder’ set to ‘iqtree’, ‘mar’, and ‘seq-recon’ set to ‘iqtree’ [58, 59] and masked with maskrc-svg v0.5 with switch ‘gubbins’ on (https://github.com/kwongj/maskrc-svg). Genomes were removed from our analysis completely if the percentage of missing data was above the 25 % cut off as determined by Gubbins. A maximum-likelihood phylogenetic tree of the masked alignment was constructed with IQ-TREE v2.2.0 and ModelFinder using model K3Pu+F+R2, 1000 ultrafast bootstraps, and 1000 bootstraps replicates for the SH-like approximate likelihood ratio test (SH-alrt) [59–61]. Trees were visualized with iTOL v6.5.5 [62]. The list of genomes used are in Table S3.

## Results

### Alberta serogroup 20 isolates are primarily ST235

**Fig. 1.**
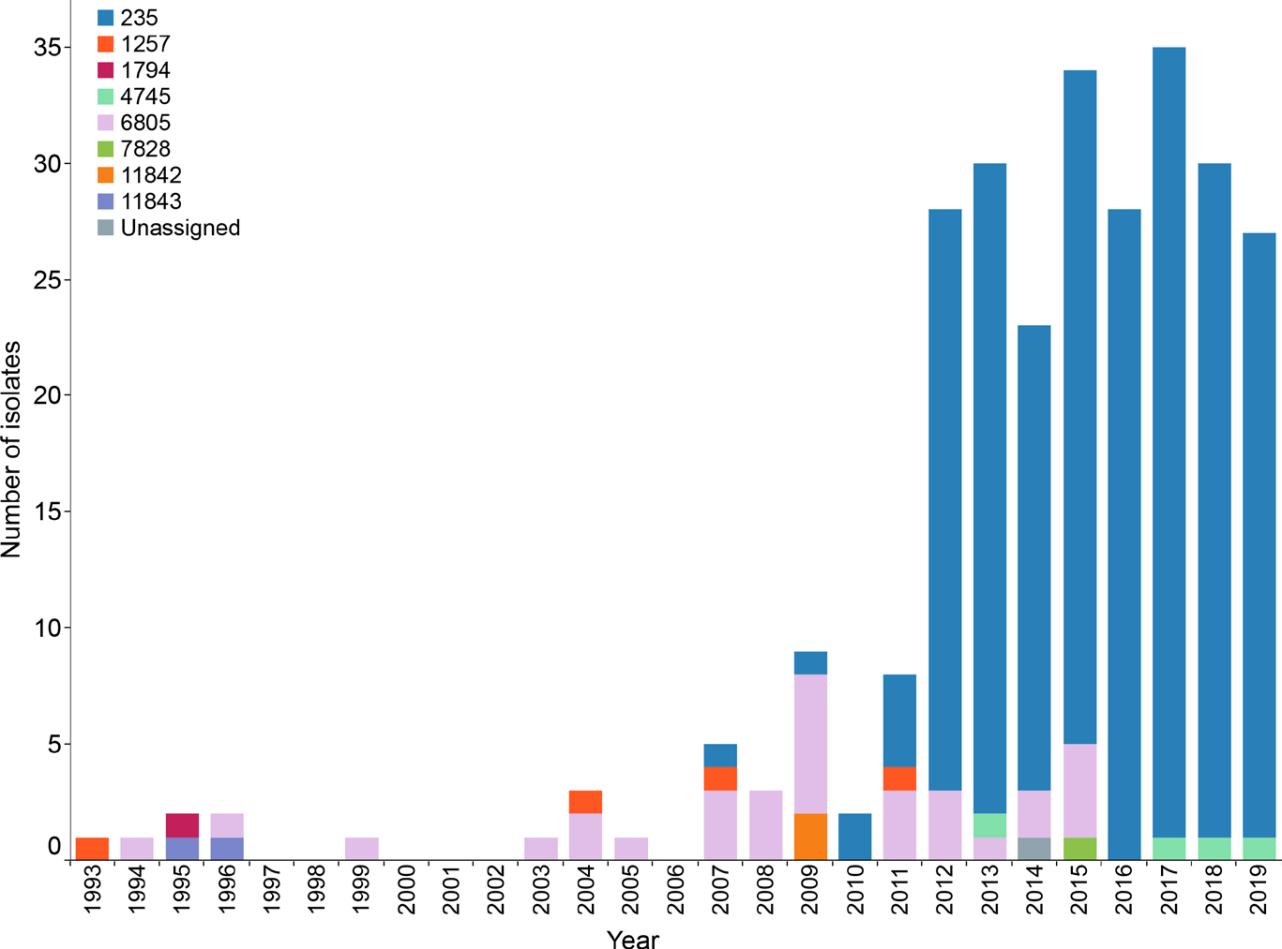
Sequence types (STs) of invasive *S. pneumoniae* serogroup 20 in Alberta over time. Stacked bars are coloured according to MLST.

From 1993–2019, 283 cases of invasive *S. pneumoniae* serogroup 20 were identified in Alberta, of which 274 were sequenced and analysed further (Table S4). These serogroup 20 isolates accounted for 2.8 %(274/9772 isolates) of all invasive *S. pneumoniae* across the entire study period but ranked in the top 10 of serotypes from 2012 to 2019. From 1993–2006, only 12 serogroup 20 cases were identified, with less than five cases per year and several years with zero cases (Fig. 1). From 2007–2019, there were 262 cases with over 20 cases per year from 2012 to 2019 and a peak of 35 cases in 2017. All isolates were categorized by *in silico* multi-locus sequence typing (MLST). Alberta serogroup 20 isolates (*n*=274) fell into eight sequence types (STs): 235 (*n*=227), 1257 (*n*=4), 1794 (*n*=1), 4745 (*n*=4), 6805 (*n*=32), 7828 (*n*=1), 11 842 (*n*=2) and 11 843 (*n*=2), with a single isolate that was not assigned an ST by PubMLST (Fig. 1; Table S4). From 1993–2011, the STs identified were STs 235 (*n*=8), 1257 (*n*=4), 1794 (*n*=1), 6805 (*n*=22), 11 842 (*n*=2) and 11 843 (*n*=2), with ST6805 being predominant (Fig. 1). From 2012–2019, ST 235 (*n*=219) was predominant, followed by STs 6805 (*n*=10), 4745 (*n*=4) and 7828 (*n*=1). Isolates were also assigned to Global Pneumococcal Sequencing Clusters, which is a typing scheme that incorporates genome-wide variation to define clusters (Table S4) [63]. The majority of Alberta isolates were part of GPSC 124 (*n*=231), which included STs 235 and 1257. The remaining Alberta isolates were part of GPSCs 43 (*n*=4; ST4745) and 61 (*n*=1; ST1794), or were part of an ST that did not have a corresponding GPSC (*n*=37; STs 6805, 7828, 11842, 11843).

### Alberta serogroup 20 were primarily blood isolates from middle-aged males

To give context to the serogroup 20 isolates collected in Alberta, body site of collection and patient age and sex was examined. Alberta strains were isolated from a variety of sterile body sites including ascitic fluid, blood, cerebrospinal fluid, chest, corneal scraping, heart valve tissue, knee, lung, pleura and synovial fluid, with blood accounting for most isolates (94.9 %; *n*=260/274) (Table S4). Among Alberta isolates, 58.4 % were isolated from male patients (*n*=160/274), 40.5 % from female patients (*n*=111/274), and 1.1 % from patients of unknown sex (*n*=3/274) (Table S4). Patients with invasive serogroup 20 infections ranged in age from <1–93 years old, with 10 patients <18 years old (50 % male, 40 % female, 10 % unknown), 36 patients 18–39 years old (72 % male, 28 % female), 136 patients 40–60 years old (60 % male, 39 % female, 1 % unknown) and 90 patients >60 years old (51 % male, 48 % female, 1 % unknown; Table S4). Most cases occurred in adults ranging in age from their late 30 s to early 70 s.

### Limited phenotypic antibiotic resistance was observed among Alberta serogroup 20 isolates

In Table 1 are the antibiotic susceptibilities of Alberta isolates, as determined by MIC and/or DD assays (data for individual isolates can be found in Table S4). Due to changes in susceptibility testing at the PPHL in Alberta over the years included in this study, not all isolates were tested with the same antibiotic panels or with the same method (i.e. MIC vs. DD). Among isolates tested, all were found to be susceptible to amoxicillin, cefepime, cefotaxime, ceftriaxone, cefuroxime, chloramphenicol, ertapenem, levofloxacin, linezolid, meropenem, penicillin/penicillin PO and vancomycin (Table 1). In addition, all Alberta isolates tested were negative for inducible clindamycin resistance. Limited antibiotic resistance was observed, with a few isolates exhibiting azithromycin (*n*=4/193 isolates tested), clindamycin (*n*=4/269), erythromycin (*n*=5/270), tetracycline (*n*=3/254) and trimethoprim/sulfamethoxazole (*n*=1/270) resistance, as well as four isolates exhibiting non-susceptibility to tigecycline (*n*=4/254; Tables 1 and S4). An intermediate phenotype was observed for three isolates to moxifloxacin and four isolates to trimethoprim/sulfamethoxazole. Interestingly, 10 isolates had conflicting results between MIC and DD assays for trimethoprim/sulfamethoxazole, with one result indicating susceptibility and the other indicating an intermediate phenotype (Table 1).

**Table 1.** Phenotypic antibiotic susceptibility of invasive Alberta *S. pneumoniae* serogroup 20

Class	Antibiotic	Test method	S	I	R	Non-S	Negative	Total no. isolates tested
β-lactams	Penicillin	DD or MIC	272					272
	Penicillin PO	MIC	61					61
	Amoxicillin	MIC	191					191
	Cefuroxime	MIC	193					193
	Cefotaxime	MIC	266					266
	Ceftriaxone	MIC	267					267
	Cefepime	MIC	193					193
	Meropenem	MIC	193					193
	Ertapenem	MIC	193					193
Folate pathway antagonists	TMP-SMX	DD or MIC	255 (265)*	4 (14)*	1			270
Glycopeptides	Vancomycin	DD or MIC	269					269
Lincosamides	Clindamycin	DD or MIC	265		4			269
	Inducible clindamycin resistance	DD					156	156
Macrolides	Azithromycin	MIC	189		4			193
	Erythromycin	DD or MIC	265		5			270
Oxazolidinones	Linezolid	MIC	254					254
Phenicols	Chloramphenicol	MIC	266					266
Fluoroquinolones	Levofloxacin	MIC	266					266
	Moxifloxacin	MIC	251	3				254
Tetracyclines	Tetracycline	MIC	251		3			254
	Tigecycline	MIC	250			4		254

*Among the 270 isolates challenged with trimethoprim/sulfamethoxazole, 255 isolates tested strictly S, 4 isolates tested strictly I, 9 isolates tested S for DD and I for MIC, and one isolate tested I for DD and S for MIC.

DD, disk diffusion; I, intermediate; MIC, minimum inhibitory concentration; Non-S, non-susceptible; PO, by mouth; R, resistant; S, susceptible; TMP-SMX, trimethoprim/sulfamethoxazole.

### Geographic distribution of Alberta and GPS project *S. pneumoniae* serogroup 20 isolates

**Fig. 2.**
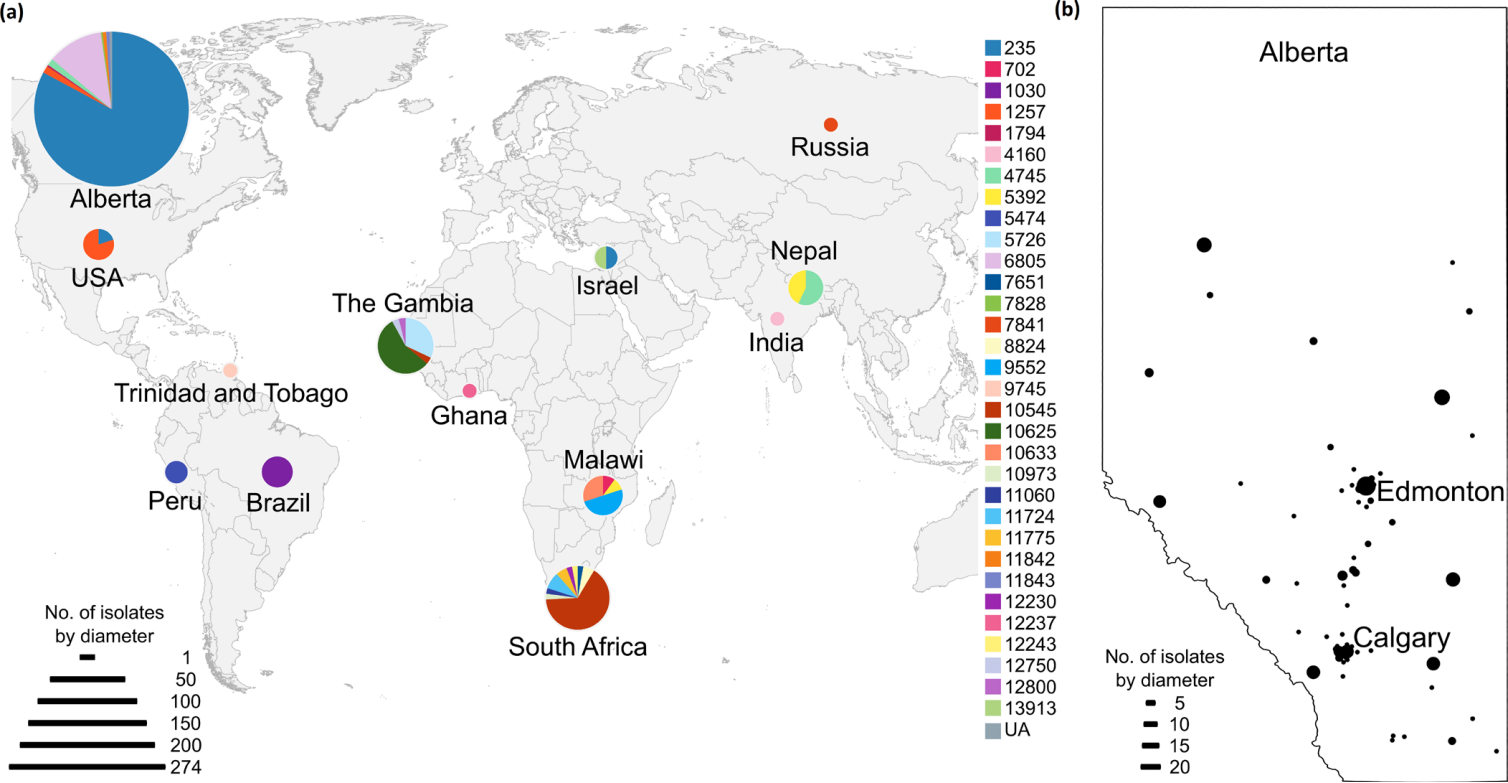
Geographic location of *S. pneumoniae* serogroup 20 isolates included in this study. (**a**) Worldwide distribution of serogroup 20 isolates from GPS Project (invasive and non-invasive isolates) and Alberta (invasive isolates). Pie charts are coloured by MLST and diameters correspond to number of isolates. Abbreviations: no., number; UA, unassigned; USA, United States of America. (**b**) Invasive serogroup 20 cases in Alberta. Dots are centred in postal code regions (first three digits) and circle diameters correspond to number of isolates. Twenty-six isolates were from individuals without an address (not shown on map).

The geographic distribution of *S. pneumoniae* serogroup 20 isolates from Alberta (*n*=274; collected 1993–2019) and the GPS Project (*n*=95; collected 1998–2015) were evaluated to position the Alberta isolates within a global context. Within the GPS Project dataset, which was comprised of primarily colonizing isolates, serogroup 20 isolates were identified in Brazil (*n*=5/95), The Gambia (*n*=25), Ghana (*n*=1), India (*n*=1), Israel (*n*=2), Malawi (*n*=10), Nepal (*n*=7), Peru (*n*=2), Russia (*n*=1), South Africa (*n*=35), Trinidad and Tobago (*n*=1), and the USA (*n*=5), representing 27 sequence types (Fig. 2a; Table S4). ST235, which was the dominant ST among Alberta isolates, was only present in the USA (*n*=1/5; 20 % of USA isolates) and Israel (*n*=1/2; 50 % of Israel isolates). The second most common ST in Alberta, ST6805, was not identified among GPS Project isolates, although the sample size for many countries was very low. Within Alberta, most isolates clustered within major cities including the capital city Edmonton (*n*=78) and Calgary (*n*=47) (Fig. 2b).

### Phylogenetic analysis of Alberta and GPS project serogroup 20 isolates

**Fig. 3.**
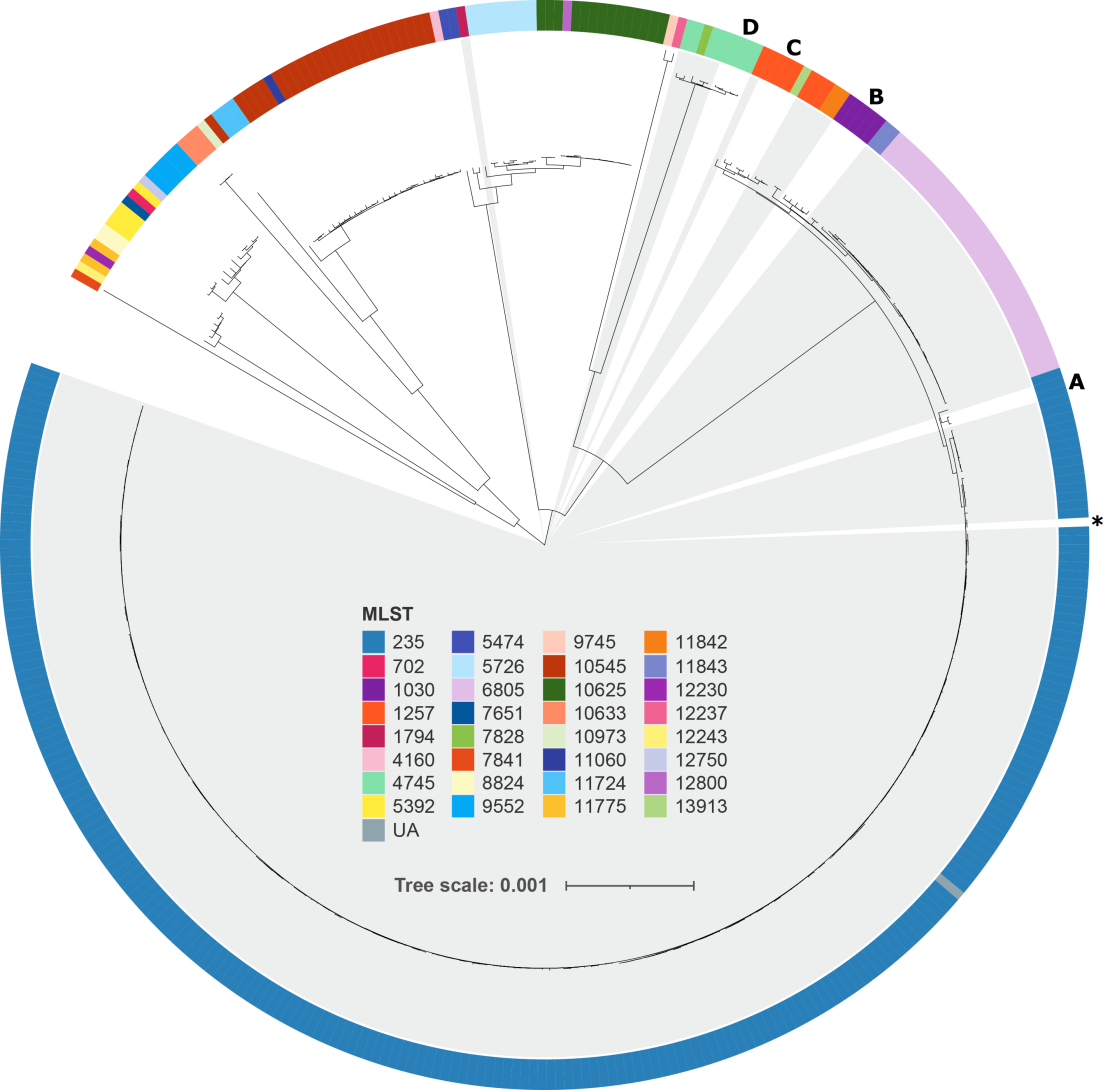
Maximum-likelihood phylogenetic tree of Alberta and GPS Project *S. pneumoniae* serogroup 20 isolates. GPS project isolates that grouped with Alberta isolates are indicated with letters: (a) one isolate from the USA (ST235; invasive) and Israel (ST235; invasive); (**b**) five isolates from Brazil (ST1030; invasive); (**c**) four isolates from the USA (ST1257; invasive) and one from Israel (ST13913; invasive); and (d) four isolates from Nepal (ST4745; one invasive, three colonizing). The reference sequence (Alberta isolate SRR3486078; ST235), used in SNP alignment generation is indicated with an asterisk (*). Branch tips are coloured by MLST and Alberta isolates are highlighted in grey. Abbreviation: UA, unassigned.

To examine the phylogenetic relationships between Alberta and GPS Project serogroup 20 isolates, a maximum-likelihood tree was generated from a core SNP alignment masked for recombination (Fig. 3). All isolates grouped primarily by ST, and most Alberta isolates clustered together (highlighted in grey in Fig. 3). Several GPS Project isolates grouped closely with Alberta isolates, which are indicated in Fig. 3. These included two ST235 isolates, one from the USA and one from Israel, that clustered with the Alberta ST235 isolates, five Brazilian ST1030 isolates that formed a sister group to the ST11843/ST6805 clade of Alberta isolates, four USA ST1257 isolates and one ST13913 isolate from Israel that clustered with Alberta ST1257 isolates, and four ST4745 isolates from Nepal that clustered within the Alberta ST4745/ST7828 clade. All the aforementioned GPS Project isolates that grouped with Alberta genomes were from cases of invasive disease with the exception of three isolates from Nepal, which were isolated via nasopharyngeal swab. One ST1794 Alberta isolate did not cluster with the other Alberta isolates but instead formed a sister group to a clade of ST5726/ST10625/ST12800 isolates from The Gambia, among which the majority were carriage (*n*=21/23 of The Gambia isolates in clade; Fig. 3). The single Alberta isolate that was not assigned a ST grouped within the Alberta ST235 clade.

### Pangenome analysis of Alberta and GPS isolates

**Fig. 4.**
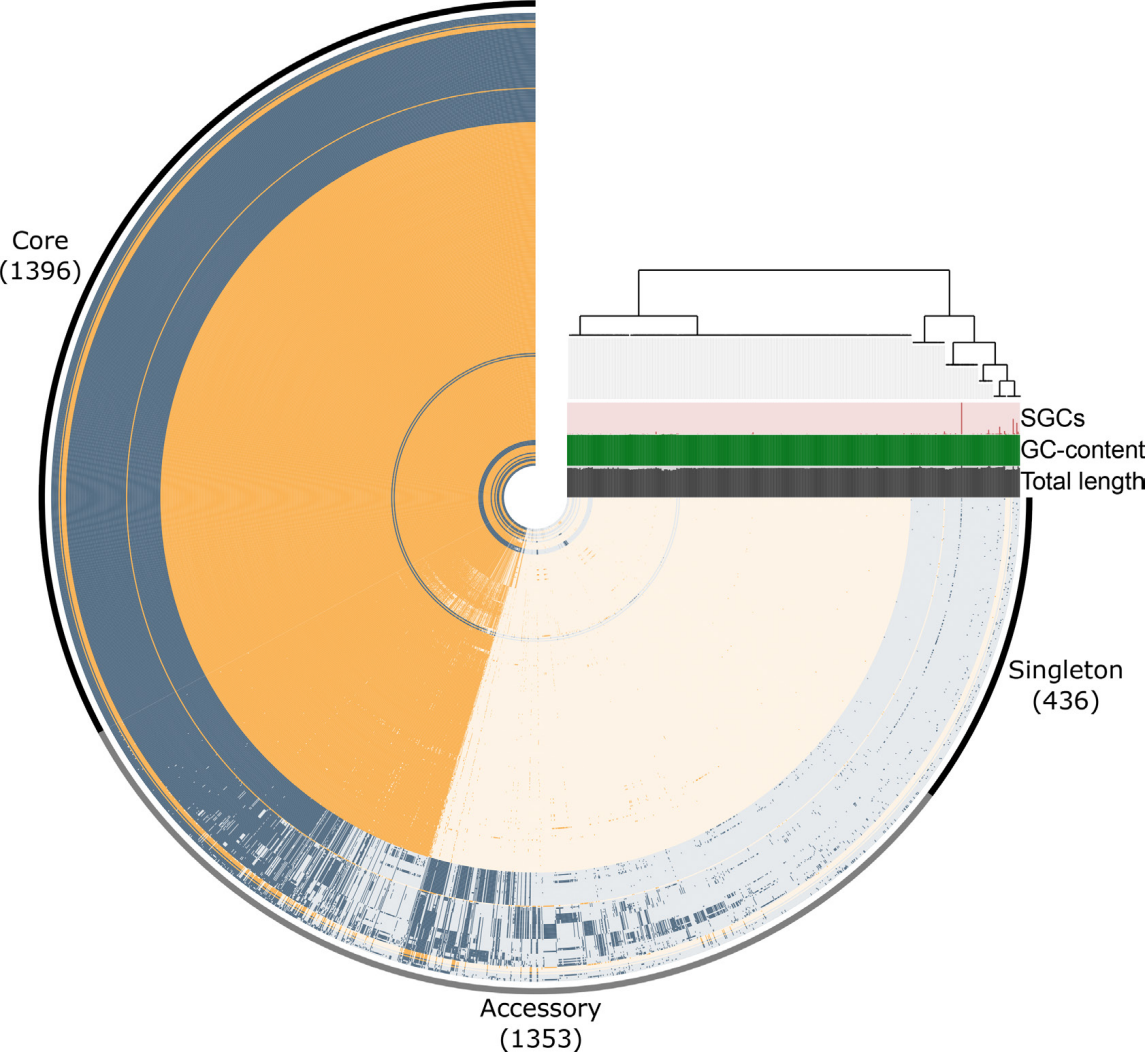
Pangenome of Alberta (yellow) and GPS Project (grey) *S. pneumoniae* serogroup 20 isolates. Each ring represents a single genome and dark orange or dark grey designates the presence of a gene. The dendrogram of isolates is ordered by fastANI. Singleton genes ranged from 0 to 132 genes, GC-content from 39.4–40.6 %, and total length from 1908068 bp–2251137 bp. Abbreviation: SGC, single gene cluster (singletons).

The pangenome of Alberta and GPS Project serogroup 20 isolates (*n*=369) comprised a total of 3185 genes (Fig. 4). The core genome contained 1396 genes present in all genomes (including paralogues), representing 44 % of the pangenome. The accessory genome (present in more than one but less than 369 isolates) contained 1353 genes (42 % of pangenome). Singletons (present in only one isolate) made up 14 % of the pangenome (436 genes). Singleton gene counts ranged from 0 to 132 genes, and most were carried by GPS Project isolates, with an ST10625 isolate from The Gambia carrying the highest number of singletons (ERR1192063; *n*=132 singletons). Among Alberta isolates, singleton genes ranged from 0 to 11 genes, with the highest numbers of singletons carried by isolates SRR3486098 (ST1794; *n*=11), SC21-2825-P (ST235; *n*=10), and SC21-2761-P (ST235; *n*=8). The total length of all genomes ranged from 1908068 to 2251137 bp, GC-content ranged from 39.4–40.6 %, and percent completion ranged from 97.2–100 %, as calculated by Anvi’o. These calculations were similar to Quast/BUSCO results, which determined genome length to range from 1889737 to 2227598 bp and percent completion to range from 95.27–97.97 % (Table S1). All isolates exhibited high average nucleotide identity (ANI) with a minimum ANI of 98.3 % among all Alberta and GPS Project isolates combined and a minimum ANI of 98.6 % among Alberta isolates alone.

### Antibiotic resistance genes carried by Alberta and GPS project serogroup 20 isolates

To determine the presence of antimicrobial resistance genes among both Alberta and GPS Project isolates, genomes were mined for antibiotic resistance ontologies (AROs) using the Comprehensive Antibiotic Resistance Database, a curated collection of peer-reviewed resistance determinants [50]. AROs were mined and categorized by the Resistance Gene Identifier (RGI) as ‘perfect’ or ‘strict’, signifying a perfect match to curated reference sequences or imperfect matches that met curated blastp bit score cut-offs and represent likely functional AMR gene variants, respectively [50]. All isolates carried genes similar to *patA*, *patB* and *pmrA*, which are transporters implicated in fluoroquinolone resistance, as well as a gene for RlmA(II), a methyltransferase involved in macrolide/lincosamide resistance (Table 2). Some resistance factors were carried by only a few Alberta and GPS Project isolates, which included fluoroquinolone-resistant *parC* and ribosomal protection protein *tetM*, which provides tetracycline resistance (Table 2). Antimicrobial resistance genes carried by only Alberta isolates included fosfomycin inactivation factor FosA4 and antibiotic target modifier ErmB, which confers resistance to macrolide, lincosamides and streptogramin antibiotics (Table 2). Antibiotic resistance genes present among only GPS Project isolates included an acetyltransferase involved in phenicol antibiotic inactivation, copies of *pbp1a* and *pbp2x* genes with mutations conferring β-lactam antibiotic resistance, and *tet(W/N/W*), which protects against tetracycline (Table 2). The RGI also identified ‘loose’ hits, which are hits that fall outside of the detection model cut-off values. Loose hits can provide detection of new AMR genes or distant homologs, but also may identify homologues and spurious matches not truly involved in antibiotic resistance. Included in the loose hits for this collection of genomes were 2015 resistance determinants that fell into a wide range of resistance mechanisms with percent identities below 88 %. Loose hits have been included separately in Table S5.

**Table 2.** Antimicrobial resistance genes carried by Alberta (AB) and GPS Project *S. pneumoniae* serogroup 20 isolates*

Resistance mechanism	Drug class	AMR Gene family	Best hit antimicrobial resistance ontology (ARO)	Min. % identity (AB min. % identity)†	Total no. hits (No. AB hits)‡
Antibiotic efflux	Fluoroquinolone	ATP-binding cassette (ABC) antibiotic efflux pump	*patA*	99.47 (99.65)	369 (274)
			*patB*	90.99 (90.99)	369 (274)
		Major facilitator superfamily (MFS) antibiotic efflux pump	*pmrA*	99.5 (99.54)	369 (274)
Antibiotic inactivation	Fosfomycin	Fosfomycin thiol transferase	FosA4	100	6 (6)
	Phenicol	Chloramphenicol acetyltransferase (CAT)	* Lactobacillus reuteri* cat-TC	99.02	1
Antibiotic target alteration	Cephalosporin; cephamycin; penam	Penicillin-binding protein mutations conferring resistance to beta-lactam antibiotics	* Streptococcus pneumoniae* PBP1a conferring resistance to amoxicillin	95.2	1
			* Streptococcus pneumoniae* PBP2x conferring resistance to amoxicillin	93.73	8
	Fluoroquinolone	Fluoroquinolone resistant *parC*	*Streptococcus pneumoniae parC* conferring resistance to fluoroquinolones	99.38 (99.51)	2 (1)
	Macrolide antibiotic; lincosamide	Non-erm 23S ribosomal RNA methyltransferase (G748)	RlmA(II)	98.82 (98.82)	369 (274)
	Macrolide; lincosamide; streptogramin	Erm 23S ribosomal RNA methyltransferase	ErmB	97.96 (97.96)	4 (4)
Antibiotic target protection	Tetracycline	Tetracycline-resistant ribosomal protection protein	*tet(W/N/W*)	68.31	1
			*tetM*	95.93 (95.93)	24 (5)

*Shown are perfect and strict Resistance Gene Identifier (RGI) hits to the Comprehensive Antibiotic Resistance Database (CARD) for 369 total strains of which 274 are from Alberta.

†The lowest percent identity of an isolate gene to the RGI hit among all isolates with that gene.

‡Excludes multiple copies if applicable and includes only perfect and strict RGI hits.

### Virulence factors carried by serogroup 20 isolates

Both Alberta and GPS Project serogroup 20 genomes were surveyed for virulence factors via comparison to the Virulence Factor Database (VFDB) core gene set, which contains representative genes for experimentally verified virulence factors [51]. Virulence factor genes carried by all isolates included those encoding multiple adherence factors (*pce/cbpE*, *pavA*), the exoenzyme neuraminidase A, the exotoxin pneumolysin, multiple capsular polysaccharide genes, and PsaA, which is involved in manganese acquisition (Table 3). Most isolates also carried adherence factor *pfbA* (*n*=347/369 isolates), as well as exoenzymes *cbpD* (*n*=273/369), *lytA* (*n*=356/369), *hysA* (*n*=367/369) and *nanB* (*n*=359/369). Surface protein A (*pspA*) was only carried by few isolates across both genome sets, including a single Alberta isolate. Factors identified exclusively among GPS Project genomes included adherence factors *cbpA*/*pspC* and *cbpG*, capsular gene *cps4B*, and IgA1 protease genes *iga* and *zmpC*.

**Table 3.** Virulence factors carried by Alberta (AB) and GPS Project *S. pneumoniae* serogroup 20 isolates*

Class	Virulence factor	Gene	Description	Min. % coverage (AB min. % coverage)†	Min. % identity (AB min. % identity)‡	No. isolates (No. AB isolates)§
Adherence	CbpA/PspC	*cbpA/pspC*	Choline binding protein A	91.74	93.6	1
	CBPs	*cbpG*	Choline binding protein G	99.64	96.24	3
		*pce/cbpE*	Choline binding protein E	97.08 (97.08)	95.06 (95.06)	369 (274)
	PavA	*pavA*	Fibronectin-binding protein-like protein A	99.94 (99.94)	99.28 (99.34)	369 (274)
	PfbA	*pfbA*	Cell wall surface anchor family protein plasminogen- and fibronectin-binding protein A	81.16 (81.16)	97.99 (99.17)	347 (252)
Exoenzyme	Autolysin	*cbpD*	Choline binding protein D	100 (100)	97.92 (98.44)	273 (217)
		*lytA*	Autolysin (N-acetylmuramoyl-l-alanine amidase)	85.79 (85.79)	84.84 (85.66)	356 (262)
	Hyaluronidase	*hysA*	Hyaluronidase	99.97 (99.97)	98.51 (99.22)	367 (273)
	Neuraminidase	*nanA*	Neuraminidase A	92.14 (92.14)	91.34 (91.34)	369 (274)
		*nanB*	Neuraminidase B	83.95 (83.95)	99.09 (99.09)	359 (264)
Exotoxin	Pneumolysin	*ply*	Pneumolysin	99.58 (99.58)	98.73 (98.73)	369 (274)
Immune modulation	Capsule	*cps4A*	Capsular polysaccharide biosynthesis protein Cps4A	99.79 (99.79)	95.44 (95.51)	369 (274)
		*cps4B*	Capsular polysaccharide biosynthesis protein Cps4B	100	80.06	44
		*cps4D*	Capsular polysaccharide biosynthesis protein Cps4D	99.85 (99.85)	94.14 (94.14)	369 (274)
		*cpsC*	Capsular polysaccharide biosynthesis protein CpsC	100 (100)	87.88 (87.88)	369 (274)
	IgA1 protease	*iga*	G5 domain-containing protein	99.58	88.12	2
		*zmpC*	Immunoglobulin A1 protease	100	99.96	1
	PspA	*pspA*	Surface protein A	87.52 (87.52)	82.97 (83.57)	16 (1)
Nutritional/ metabolic factor	PsaA	*psaA*	Manganese ABC transporter manganese-binding adhesion lipoprotein	100 (100)	98.71 (99.36)	369 (274)

*Shown are Virulence Factor Database (VFDB) hits for 369 total strains of which 274 are from Alberta.

†The lowest percent sequence coverage of an isolate gene to the VFDB gene among all isolates with that gene.

‡The lowest percent identity of an isolate gene to the VFDB among all isolates with that gene.

§Excludes multiple copies if applicable.

### Alberta and GPS Project isolates are primarily serotype 20B

The serotypes of Alberta and GPS Project serogroup 20 isolates (*n*=369 total) were confirmed *in silico* by analysing capsular polysaccharide (CPS) gene clusters using PneumoCaT [47]. Among all isolates analysed, 304/369 were clearly serogroup 20, while the remaining isolates were most similar to serogroup 20 but did not meet PneumoCaT confidence cut offs. To determine if a particular serotype was dominant among serogroup 20 isolates (i.e. serotype 20A or 20B), blast was used to identify isolates with a full-length *whaF* gene, which would be suggestive of serotype 20B [10]. There were 253/369 isolates (69 %) that carried full-length *whaF* genes suggestive of serotype 20B, which included 235/274 (86 %) of the Alberta isolates. The remaining Alberta and GPS Project isolates had truncated *whaF* genes, including 31 isolates where the gene sequence was on a contig end, inhibiting the ability to determine the true length. The *whaF* gene was not identified in six isolates.

## Discussion

In this study we characterized 274 invasive *S. pneumoniae* serogroup 20 isolates identified in Alberta from 1993 to 2019 and compared these to 95 invasive and non-invasive serogroup 20 GPS Project isolates collected from 1998 to 2015. The goal was to characterize this increasingly prevalent serogroup within Alberta primarily through genomic analyses, which has not been conducted previously for this serogroup on as large a scale, to the best of our knowledge. We evaluated basic demographics, STs, *in silico* serotyping, antimicrobial resistance, virulence factors, and phylogenetic and pangenomic relationships between isolates.

In this study, the majority of Alberta serogroup 20 isolates were part of MLST 235 (ST235), which was first identified in the province in 2007. In PubMLST, 503 isolates were labelled as serotype 20, with representative isolates from Africa, Asia, Europe, North America, South America and Oceana, including 35 countries not represented in our dataset (https://pubmlst.org/; accessed 3 May 2023). Among the serogroup 20 isolates, 45 were designated ST235 (https://pubmlst.org/; accessed 3 May 2023). Source data was only available for 10/45 serogroup 20 ST235 isolates, with the majority (8/10, 80 %) being considered invasive. The second most common ST in the Alberta serogroup dataset, ST6805, had only a single isolate of representation among serogroup 20 in PubMLST, which was an invasive isolate from New Zealand (PubMLST id:13791). Among all ST235 in PubMLST (*n*=52), the majority were serogroup 20, with one isolate being serotype 7C and six with inconclusive serotyping. Interestingly, ST235 was limited among the GPS Project genomes included in this study, with single isolates in Israel and the USA that clustered phylogenetically with Alberta ST235 (Figs 2a and 3). Identification of invasive serogroup 20 ST235 in the literature is also scarce. A study from Brazil surveyed serotypes and genotypes of invasive *S. pneumoniae* before and after PVC10 implementation, finding that serogroup 20 had increased post-vaccine introduction [64]. Interestingly, these isolates were all from ST8889, which is a single locus variant of ST235 [64]. Similar to our study, the majority of isolates were from males with average and median ages of 61.6 and 62 years, respectively, and were recovered primarily from blood [16]. Clinical data was collected for the presented patients and all were found to have at least one underlying condition, with the most common being chronic obstructive pulmonary disease, alcoholism, systemic arterial hypertension and smoking, which are well-understood to facilitate pneumococcal disease [16]. Interestingly, isolate SC21-2726-P was not assigned an ST, indicating that perhaps this isolate represents a novel ST or one that is not present in the current MLST scheme. Alternatively, a lack of ST assignment may be the result of missing relevant sequences due to limitations in sequencing/assembly of the genome.

The appearance of a limited number of serogroup 20 ST235 identified outside of Alberta may be due to a variety of factors. First, if ST235 is associated with invasive disease, representation within the GPS Project may be limited since most isolates were not invasive. Second, the GPS Project sample size was small, isolates were collected over a slightly different time frame than Alberta isolates (1993–2019 for Alberta vs. 1998–2015 for GPS), and isolates had a limited global representation of only 12 countries. If ST235 has had a more recent or globally restricted emergence, it is possible that inclusion of a larger number of global genomes over an extended time frame would reveal additional ST235 isolates and shed more light on the profile of this ST globally. Finally, limited distribution may be due to the overall low incidence of ST235 among *S. pneumoniae* isolates as suggested by the relatively small number of ST235 in PubMLST. As we do not have representative global data and are limited by sample size, additional study and surveillance would be required to ascertain the true global distribution of ST235.

Rates of antibiotic resistance are increasing among *S. pneumoniae* isolates and vary depending on geographic location [65]. Therefore, tracking antimicrobial resistance can provide insight into local, national and international rates and types of resistance. In general, resistance has been observed for multiple antibiotics, including β-lactams, macrolides, fluoroquinolones, trimethoprim-sulfamethoxazole (TMP-SMX) and tetracyclines. Antibiotic resistance was limited among the Alberta *S. pneumoniae* serogroup 20 isolates in this study, with ≤2 % resistance among isolates challenged with azithromycin, clindamycin, erythromycin, tetracycline and TMP-SMX (Table 1). In 2020, a Canadian Communicable Disease Report publication on invasive pneumococcal disease across Canada reported 45 serogroup 20 isolates, among which the following antibiotic resistance was observed: 4.4 % to clarithromycin, 2.2 % to clindamycin, 6.7 % to doxycycline, 6.7 % to TMP-SMX and 2.2 % multi-drug resistance [66]. These percentages were below the already low national percentages for all serotypes combined for 2020. No resistance was reported for serogroup 20 to penicillin, ceftriaxone, imipenem, meropenem, levofloxacin and chloramphenicol in the national study. Among the antibiotics tested in both our study and included in the national survey, similar antibiotic resistance rates were observed with the exception of clindamycin and TMP-SMX, which had higher percent resistance in the national study (Table 1).

Serogroup 20 isolates carried several antibiotic resistance genes (Table 2). All isolates included in our study carried *patA*, *patB* and *pmrA* efflux pumps, which can confer fluoroquinolone resistance, as well as RlmA(II), a methyltransferase that can confer resistance to tylosin and other mycinosylated macrolides (Table 2) [67]. The efflux pump *pmrA* has been shown to impart low-level resistance to norfloxacin but does not appear to play a major role in fluoroquinolone resistance in *S. pneumoniae* [68, 69]. Resistance to hydrophilic fluoroquinolones (e.g. ciprofloxacin, norfloxacin) appears to be linked to overexpression of *patA/patB*, and the presence of these genes within wild-type strains confers only low-level intrinsic resistance [70]. The Comprehensive Antibiotic Resistance Database provides prevalence data for antibiotic resistance genes, and identified *patA*, *patB*, *pmrA* and RlmA(II) in 99.49 %, 100 %, 94.36 % and 99.49 %, respectively, of complete *S. pneumoniae* chromosomes in NCBI as of 23 June 2022 (https://card.mcmaster.ca/home accessed 13 May 2023) [71]. Thus, is it not surprising to see the universal carriage of these genes among the *S. pneumoniae* serogroup 20 characterized in this study, as carriage appears high among *S. pneumoniae* in general. The remaining antibiotic resistance genes identified had very limited distribution, including among Alberta serogroup 20 isolates, which is not surprising given the limited phenotypic resistance observed (Table 2).

The Alberta and GPS Project genomes were characterized further in terms of genomic composition. All isolates had Average Nucleotide Identity (ANI) values above 98 %, indicating their close genomic relatedness. Similarities were also observed for virulence factor carriage, which was comparable across all isolates, with most virulence factors being present in all isolates (Table 3). No distinct patterns emerged linking virulence factors to the invasive Alberta isolates in comparison to the predominantly non-invasive GPS Project isolates. Interestingly, the serogroup 20 isolates carried a high number of accessory and singleton genes. This observation aligns with the notion that *S. pneumoniae* has an ‘open’ genome, whereby the inclusion of more isolates would contribute an additional, albeit decreasing, number of genes in the form of non-core genes [72]. Notably, the Alberta isolates carried the fewest singleton genes, potentially due to the clonal nature of these isolates, particularly those belonging to the ST235 lineage (Fig. 4).

Currently, PPV23 is the only vaccine that contains serogroup 20. Previous studies have determined that PPV23 contains serotype 20A polysaccharide and that antibodies from PPV23 vaccination mediate opsonophagocytic killing of serotype 20B *in vitro*; nevertheless, the clinical relevance of this cross-reactivity requires further epidemiological investigation [10, 11]. In Alberta, despite the implementation of PPV23 vaccination in 1997, cases of invasive serogroup 20, appearing to be predominantly serotype 20B, have been increasing, potentially calling into question the cross-protectivity of the vaccine. However, as detailed clinical and socioeconomic data was not available for the cases presented in this study, it is possible that the increase in cases is due to transmission among vulnerable groups that have not been previously vaccinated. In Alberta, the increase in case numbers has been primarily among middle-aged males, who would not necessarily be eligible under current vaccination programme criteria. We also saw clustering in cities, which could be an indicator of increased risk within these areas (Fig. 2b). For example, serogroup 20 has been found to be disproportionately represented among homeless individuals in comparison to the general population, and in general, IPD in this group has highest incidence among middle-aged males [73]. If this is the case in Alberta, targeted vaccination programmes for at-risk individuals using serogroup 20-containing vaccines such as the PPV23, or when available the conjugate vaccine V116, may be necessary if case numbers continue to increase.

## Conclusion

The increasing prevalence of invasive *S. pneumoniae* serogroup 20 in Alberta underscores the need for ongoing surveillance and identification of high-risk population groups. This will require continued surveillance of serogroup 20 both locally and globally to better understand epidemiological trends, develop effective prevention and control strategies, and contribute to pathogen characterization. Genomics has emerged as a valuable tool in pathogen characterization and epidemiology, enabling better understanding of the spread and genetic characteristics of this pathogen. By leveraging genomics-based epidemiological studies to inform public health strategies it is hoped that the burden of pneumococcal disease can be minimized.

## Supplementary Data

Supplementary material 1
